# Symptomatology of posttraumatic stress and burnout associated with aggressions suffered by primary care health professionals. A transversal study

**DOI:** 10.3389/fpsyg.2024.1335155

**Published:** 2024-06-26

**Authors:** Santiago Gascon-Santos, Bárbara Oliván-Blázquez, Maria José Chambel, Lucía Sebastián, Adrián Alacreu-Crespo, Yago Pérez-Montesinos, Pilar Paúl, Ricardo Fueyo-Díaz

**Affiliations:** ^1^Department of Psychology and Sociology, University of Zaragoza, Zaragoza, Spain; ^2^Research Team PROSAM Aragon Government, Zaragoza, Spain; ^3^Aragon Health Service, Institute for Health Research (IIS Aragon), Zaragoza, Aragon, Spain; ^4^Department of Social Psychology, University of Lisbon, Lisbon, Portugal; ^5^Department of Psychology and Sociologý, University of Zaragoza, Zaragoza, Spain; ^6^Hospital Universitario Miguel Servet, Zaragoza, Spain

**Keywords:** aggressions, primary care, Posttraumatic Stress Disorder, burnout, psychological damage

## Abstract

**Objective:**

To know the incidence of aggressions in Primary Care, and to determine the psychological symptoms that may accompany these experiences. A transversal study was carried out in North-Eastern Spain, in 2022.

**Methods:**

207 participants (60.9% women, mean age 48.8 years), primary care professionals (nurses and doctors), responded to Questionnaire of Sociodemographic and Occupational variables, List of Aggressions, General Health Questionnaire, Posttraumatic Stress Disorder Checklist, and Maslach Burnout Inventory.

**Results:**

57.49% had suffered aggressions in the last year (44.4% threats, 55.1% insults and 18.4% physical aggressions). They showed more psychological symptoms than those who had not been assaulted: re-experiencing, avoidance, distancing, emotional or cognitive disturbances and hypervigilance, as well as to emotional exhaustion, depersonalization and low personal realization. Although no statistically significant differences were found in terms of the number of victims with respect to gender, men showed more symptoms of trauma.

**Conclusion:**

The data show the increase of violent episodes in Primary Care. Although the attacks perpetrated in this sector do not usually present the magnitude required for the diagnosis of post-traumatic stress, many of its symptoms were manifested in those who had suffered violence. Likewise, a relationship was found between aggression and burnout. The need to have post-incident protocols is evident, raising awareness among professionals about the importance of reporting episodes, as well as designing and implementing prevention plans. The damage generated by these behaviors does not only affect nurses and doctors, but, indirectly, all users of the health system.

## 1 Introduction

Violence is a phenomenon present in numerous interpersonal actions, including the work environment. The International Labour Organization defines workplace violence as “any action, incident or behavior by which a person is assaulted, threatened or humiliated in the course of or as a consequence of his or her work” (Di Martino, 2003). The Health Division of Safety Research (1996) distinguishes between different types of workplace violence based on the relationship between aggressors and victims and classifies as type I when the aggressor is not related to work; type II when the aggressor is the recipient of a service (students, patients, clients, etc.) and type III when the aggressor and victim have a work relationship (boss, co-worker).

Research has focused on episodes of physical aggression, as it is believed that its consequences for health could be the most serious. In recent decades, more and more attention has been paid to verbal violence, differentiating between insults and threats (Rudkjoebing et al., 2020). Winstanley and Whittington (2004) cataloged these incidents in order to avoid confusion and define physical aggression as the use of force against another person, producing physical, sexual or psychological harm (punching, slapping, pushing, even the use of weapons), distinguishing it from threatening behavior–which involves a declaration of intent to cause harm, provoking fear in the target–and from verbal abuse (insults, teasing, etc.) as an action that humiliates or implies lack of respect for dignity.

The professions most affected by interpersonal violence are those in the service sector. Police officers, security guards or healthcare professionals are the main victims of aggression in the workplace (Menckel and Viitasara, 2002). As a result, this issue has become a matter of concern for international organizations, governments and professional associations. The World Health Organization has estimated that 25% of violence in the workplace corresponds to the healthcare sector, and that more than half of its professionals have suffered some episode of this type in the course of their work. These figures seem to be increasing according to recent studies (Mento et al., 2020).

Previous research concluded that assaults were more frequent and severe in large hospitals–specifically in emergency and psychiatric departments–while much lower numbers of incidents were significantly reported in smaller institutions and primary care centers (Gascón et al., 2009).

In recent decades, in different countries, there has been a healthcare collapse, especially in public primary care services, due to socio-economic reasons as well as to the emergency generated by the COVID-19 pandemic (Eurofound-ETF, 2022). It is reported that there is a greater burden of care and an increase in waiting times, which contributes to generate discomfort in professionals and patients, propitiating the conditions for violent episodes to occur (Moleras-Serra et al., 2023).

In Spain, incidences in this sector have increased to reach figures close to 50% of assaulted healthcare workers in the twelve months prior to self-reporting, and affecting up to 75% over the course of their professional life (Bernaldo-De-Quirós et al., 2005). These data come from research studies, as official figures are based exclusively on complaints–and these only reflect the most serious cases of physical attacks that have required medical attention and account for between 2 and 3.7%–while mild cases, threats and insults are rarely reported. Healthcare professionals may not report for various reasons, either because they end up “normalizing” these behaviors, or because they do not feel supported by their organization (Spelten et al., 2020).

Despite its increasing incidence, studies on this phenomenon have been limited and it is only now that publications on the contribution to possible psychological harm in those who suffer from it are increasing (Chirico et al., 2022), which is considered a priority with a view to developing preventive programs (Lanctôt and Guay, 2014).

### 1.1 Objective

In view of the above, the objective was to determine the incidence of aggressions in primary care centers, considering all types of violence perpetrated by users, as well as to contrast the possible associated symptomatology in nurses and doctors who had suffered this type of episode with those who had not.

### 1.2 Theoretical framework

Abundant literature supports the relationships between stress and health. From the interactive theories of stress, great importance is attributed to chronic stress and daily events that involve discomfort, worry or discomfort, which are related to both physical health and anxiety and mood disorders (Lanctôt and Guay, 2014).

Beyond interactive theories, Post Traumatic Stress Disorder (PTSD) can manifest when the person has lived through or witnessed a shocking event, such as threat of death, violence or injury. The symptoms described (American Psychiatric Association, 2013) are re-experiencing, avoidance, cognitive or emotional disturbances, and arousal and hypervigilance. Although PTSD was initially studied in survivors of war and major catastrophes, in recent decades the spectrum has been broadened to experiences such as sexual abuse, family or workplace violence (Rudkjoebing et al., 2020). More specifically, several studies have found links between assaults on health professionals and PTSD-related symptomatology, such as anxiety, irritability, insomnia, or depression (Varghese et al., 2022).

On the other hand, in the relationship between work stress and health, we should pay attention to the burnout phenomenon, constituted by the factors of emotional exhaustion (EE), depersonalization (DP), and lack of realization by work (RP) (Maslach and Leiter, 2016). To understand the different genesis of this syndrome, Maslach and Leiter (2008) proposed a two process model of burnout, which states, in summary, that while work overload acts directly on the EE (producing professionals to experience fatigue due to excessive demands and lack of time to recover), a second process can occur, in which overload is not reflected in high levels of EE because other variables–such as control, rewards, feelings of community or justice–act as protectors, through a congruence between the values of the individual and the values of his or her organization; thus, a change in the negative direction of these values could trigger an acceleration of the burnout process.

In occupations that require a high degree of demands -such as nurses and doctors- it is common to observe not only exhaustion due to excessive workload, but also a loss of involvement and a feeling of accomplishment for the work. Thus, in environments where aggressions are common, it has been found a decreased sense of control, a negative perception of rewards and, in the case of not finding support, absence of sense of community and justice, all of which erodes the possible congruence of values and commitment necessary to perform care tasks (Viotti et al., 2015).

Similarly, many studies have confirmed relationships between the permanent risk of experiencing violence, regardless of the form or intensity of the episodes, and dimensions of burnout (Gascón et al., 2013; Kind et al., 2018).

Finally, several authors note that physical aggressions do not have as serious consequences for the psychological health of the victims as might be expected, while a hostile climate with continuous insults and threatening behavior could have worse consequences, especially in terms of symptoms constituting PTSD, anxiety, depression, or negative indices in the dimensions of burnout (Kobayashi et al., 2020; Rudkjoebing et al., 2020). The association between physical violence and psychological symptomatology has sometimes yielded contradictory results, with some studies finding that verbal aggressions, insults and threats could have a greater impact on mental health than physical assaults themselves, while other research showed that physical aggressions are often accompanied by verbal violence and that both types showed a negative relationship with various symptoms of stress, anxiety and burnout (Bernaldo-De-Quirós et al., 2005; Kind et al., 2018).

The following hypotheses were established for this study:

Hypothesis 1: Those who have suffered some kind of aggression will show a higher rate of psychological symptoms than those who have not suffered aggression.

Hypothesis 2: Those who have been victims of non-physical aggression, such as insults and threats, will report a higher number of symptoms than those who have suffered physical violence.

## 2 Materials and methods

The study was carried out between September and December 2022 in the three provinces of N.E. Spain, where 1,988 healthcare professionals (doctors, nurses and other professionals) provide services. Given the observed phenomenon of underreporting, it was impossible to establish a priori two groups of professionals according to whether or not they had been victims of attacks. For this reason, the Department of Health of the Government was asked for advice in order to select a number of centers that would provide a representative sample. Subsequently, this department sent information and an invitation to participate to 836 professionals from 19 primary care centers (urban and rural). Based on previous data (Gascón et al., 2013), with a confidence level of 95%, a precision of 5% and considering that 11% of professionals have suffered physical aggression, the required sample size was 146 participants (to which 10% was added).

### 2.1 Participants

A total of 244 professionals showed their willingness to participate in the study. After applying the aforementioned criteria, 207 were considered for the study (participants in an active work situation, without sick leave). The inclusion criteria were: (a) being over 18 years of age, (b) not suffering from a mental disorder, (c) giving informed consent and (d) providing services in primary care. Lack of consent prevented access to the questionnaire and incomplete surveys were not included.

### 2.2 Variables and instruments

In addition to socio-demographic and work-related variables (gender, age, profession, position, seniority, family cohabitation), variables related to aggressions (number, types of violence, reporting, feeling of being supported by the organization), variables related to PTSD (re-experiencing, avoidance, cognitive or emotional disturbances, arousal and hypervigilance) and burnout symptoms (emotional exhaustion, depersonalization, lack of personal realization by work) were analyzed through the following questionnaires:

Sociodemographic and labor variables: Participants completed a demographic and labor data form with information on age, gender, family situation, profession, work shift, seniority and administrative situation.

List of assaults: Violence Type II -exercised by users, patients or companions-. Each item describes a type of aggression: physical, threats, threatening behavior and verbal abuse (Winstanley and Whittington, 2004) and is answered on a Likert scale from 0 (never), 1 (on one occasion) and 2 (on more than one occasion). The fact of having been attacked, or not having suffered aggression, was used as a dichotomous variable for contrast between groups. In relation to this, they answer if they have denounced or reported the episode and if they feel, or not, support from their management in cases of violence.

Psychological symptomatology: Evaluated by means of the General Health Questionnaire (GHQ-12), whose 12 items measure somatic symptomatology, depression, anxiety and insomnia and dysfunction in the social area. It is answered on a Likert-type scale, where 0 represents always and 3 never. The validated version for the Spanish population showed Cronbach’s alpha indices between 0.74 and 0.81 (Sánchez-López and Dresch, 2008). In this study, the global measure was used as an index of possible symptomatology and obtained an alpha of 0.88.

Posttraumatic stress symptoms: Assessed using the Posttraumatic Stress Disorder Checklist for DSM-5 (PCL-5, Weathers et al., 2013), composed of 20 items that are grouped in terms of constituent PTSD symptomatology experienced in the past month and scored on a Likert-type scale whose score ranges from 0 (not at all) to 4 (totally); for example, “how much have you been bothered by repeated, disturbing, and unwanted memories about the event?” The scores generate four subscales: re-experiencing REE, avoidance AV, cognitive or emotional disturbances CED, and arousal and hypervigilance AH. The Spanish version obtained an α = 0.94 to 0.96 (Soberón et al., 2016), and, in the sample of this study, an α = 0.93.

Burnout dimensions: The Maslach Burnout Inventory-General Survey (MBI-GS) was used, offering information on the dimensions of emotional exhaustion (EE), depersonalization (DP) and personal realization by work (PR). Its 16 items are answered by means of a Likert scale expressing the frequency in which a situation has been experienced; for example, the statement “*I feel emotionally exhausted at work*” can be answered from 0 (never) to 6 (daily). Low levels in RP and high rates in EE and DP indicate a greater experience of job burnout. In different studies, using the version validated for the Spanish population, reliability indices above 0.74 have been found in all three dimensions (Salanova et al., 2000). In the present study, the indices were 0.81 in EE, 0.76 in DP and 0.70 in RP.

### 2.3 Ethical aspects

The project was approved by the Clinical Research Ethics Committee of Aragón (CEICA, PI22/049). The procedures carried out complied with ethical standards and with the 1975 Declaration of Helsinki. The professionals were informed that their participation was voluntary and that the responses obtained would be treated anonymously and confidentially. All of them signed an informed consent form and were subsequently informed of the results.

### 2.4 Statistical analysis

The use of parametric tests was considered appropriate, given the sample size. For the description of the characteristics of the sample, frequencies and percentages were used for categorical variables and means and SDs for continuous variables. In the bivariate analysis of comparison between professionals who have suffered aggressions and those who have not, the Chi-square statistic was used to compare categorical variables and Student’s *t*-test to compare continuous variables.

Although a cross-sectional survey design was used, both because of the type of population and the subject matter about which participants were asked, it is considered unlikely that factors such as the consistency effect, halo effect or social desirability, have acted to produce bias.

An exploratory analysis with all the variables of interest resulted in a matrix of two factors, one of them related to the dimensions of burnout, and the other to the symptomatology of post-traumatic stress disorder, which explain the variance equally (Hair et al., 2006), so it was considered that the results would not show a possible bias due to the variance of the common method.

Finally, multivariate logistic regression analyses were performed to establish those factors that could be predictive of aggressions, introducing in the model, as a dependent variable, having been a victim of aggression and, as predictive variables, sociodemographic and work-related variables. Subsequently, multivariate analyses (stepwise linear regression) were performed to predict those variables (sociodemographic, occupational and aggressions) that may contribute to PTSD symptomatology and burnout dimensions.

## 3 Results

The profile of the participant was a man or woman with an average age of 48.79 years, living with family members, working in ordinary care, as a doctor or nurse, with a seniority of almost 20 years, with an interim or permanent contract. Regarding aggressions, more than half (57.49%) of the participants had suffered them, being mainly threats (44.41%) or insults (55.14%), and physical aggressions (18.35%). Of these, only 18 (15.1% of those assaulted) recorded the incident and only one had filed a complaint. A total of 80.1% of the professionals doubted that their organization would support them in the event of a complaint (Table 1).

**TABLE 1:** Description of the sample in the variables collected (sociodemographic, occupational, psychological symptomatology, burnout, PTSD, as well as incidents recorded).

Variables	Frequency (%) Mean (DT) *N* = 207	No aggressions *N* = 88	Aggressions *N* = 119	χ^2^.*P*-value 30.94 (0.000)
**Sex**				
Man	81 (39.1%)	34 (41.9%)	47 (58.1 %)	0.016 (0.900)
Woman	126 (60.9%)	54 (42.8%)	72 (57.1%)	
Age	48.79 (9.51)	49.11 (10.54)	48.55 (8.68)	
**Convivience**				
No family coexistence	70 (33.8%)	24 (34.3%)	46 (65.7%)	2.929 (0.087)
With family living	137 (66.2%)	64 (46.7%)	73 (53.2%)	
**Type of care**				
Routine care	165 (79.7%)	78 (47.3%)	87 (52.5%)	5.743 (0.017)
Continuous attention	42 (20.3%)	10 (23.8%)	32 (76.2%)	
**Profession**				
Doctor	101 (48.8%)	43 (42.6%)	58 (57.3%)	1.586 (0.452)
Nursing	97 (46.9%)	42 (43.3%)	55 (56.6%)	
Others	9 (4.3%)	2 (22.2%)	7 (77.7%)	
Seniority	19.90 (10.00)	20.58 (10.98)	19.39 (9.20)	
**Situation**				
Temporary contract	25 (12.1%)	13 (52%)	12 (48%)	4.303 (0.116)
Interim	63 (30.4%)	20 (31.7%)	43 (68.2%)	
Permanent	119 (57.5%)	55 (46.2%)	64 (53.7%)	
**Frequency aggress**				
No	88 (42.5%)	88		207.000 (0.000)
One aggression	29 (13.5%)		29	
More than one aggression	90 (43.9%)		90	
**Type of aggressions**				
Physical aggression	38 (18.4%)			
Threats	89 (44.4%)			
Insults	113 (55.1%)			
**MBI**				
Emotional exhaustion (EE)	59 (28.5%)	13 (14.7%)	46 (38%)	1.839 (0.022)
Depersonalization (DP)	69 (33.3%)	10 (11.3 %)	49 (42.2 %)	2.107 (0.008)
(Lack) Personal realization (PR)	75 (36.2%)	21 (23.8 %)	54 (55.4%)	2.724 (0.043)
**PTSD**	46 (21.8%)	5 (5.7%)	41 (34.5%)	1.005 (0.001)

Although the authors of the Burnout Questionnaire (MBI-GS) are not in favor of offering cut-off points and have repeatedly advised against their use, since the questionnaire offers dimensional measures in three variables (EE, DP, RP) that make up burnout syndrome cannot be dichotomous, but dimensional (Maslach and Leiter, 2008), in the present study we have used the criterion that participants’ measurements were in the fourth quartile to establish their symptomatology in the three dimensions of the syndrome (Schaufeli et al., 2001; Brenninkmeijer and Van Yperen, 2003). In this way, the difference between the sub-samples who report aggression and those who do not can be observed in terms of the dimensions of burnout (Table 1).

On the other hand, although PTSD is a pathology resulting from having lived through or witnessed a traumatic situation, or from repeated exposure to details of traumatic events (Echeburúa, 2017) and, in order to be diagnosed, as a first requirement, such an event must have occurred. The episodes of violence commonly reported in Primary Care do not have the characteristics that may accompany acts of war, situations of major catastrophes, rape, etc. However, the hypothesis was established that, perhaps, the victims of this daily violence could show some of the symptoms of re-experiencing, avoidance attempts, cognitive or emotional problems, distancing or hypervigilance, required when diagnosing PTSD.

The aim was rather to establish a dimensional measure of possible trauma, from a research perspective, rather than to obtain a diagnosis, for which a clinical interview would be much more advisable. The Posttraumatic Stress Disorder Checklist for DSM-5 (PCL-5 is an undoubtedly helpful tool, especially for recording the constituent symptomatology of this disorder). Although it was not designed to make an “all or nothing” diagnosis, in Table 1 shows the sum values of the subscales that make up the PCL-5 and takes as a cut-off point those that are above 33 points (Durón-Figueroa et al., 2019).

Through a contrast of means (*t*-test), the symptomatology variables studied, with the exception of general health, showed statistically significant differences between professionals who had not suffered aggressions and those who had experienced them, both in PTSD constituent symptoms, and in the burnout dimensions (Table 2).

**TABLE 2:** Contrast of means (*t*-test) in the psychological variables (victims-non-victims).

	Aggressions						
	No M (DS)	Yes M (DS)	*T*	Sig (bilat)	Dif aver	Interval	
General health	25.84 (5.72)	27.05 (5.22)	−1.550	0.123	−1.20	−2.727	0.327
REE	2.63 (3.82)	5.35 (5.03)	−4.421	0.000	−2.718	−3.930	−1.506
AV	1.30 (2.02)	2.84 (2.89)	−4.468	0.000	−1.527	−2.201	−0.853
ECD	1.38 (2.34)	4.03 (4.22)	−5.731	0.000	−2.643	−3.553	−1.734
DIST	1.85 (2.56)	3.07 (3.23)	−3.014	0.003	−1.214	−2.008	−0.420
AH	4.29 (3.82)	8.18 (5.50)	−5.378	0.000	−3.886	−5.311	−2.461
EE	17.15 (7.31)	23.77 (7.80)	−6.271	0.000	−6.625	−8.709	−4.542
DP	8.12 (3.03)	9.86 (2.90)	−4.170	0.000	−1.741	−2.564	−0.917
PR	7.02 (3.53)	8.98 (3.29)	−4.040	0.000	−1.944	−2.893	−0.995

REE, re-experiencing: AV, avoidance; ECD, emotional and cognitive disturbances; DIST, distancing; AH, arousal, hypervigilance; EE, emotional exhaustion; DP, depersonalization; RP, lack of personal realization by work.

Both the variables relating to diagnostic criteria for PTSD and those relating to the variables constituting burnout syndrome showed statistically significant differences between the group of victims and those who did not report aggressions. As can be seen in Table 2, the statistical significance was: REE = *t*_(205)_ −4.421. *p* ≤ 0.001; AV = *t*_(205)_ −4.468. *p* ≤ 0.001; ECD = *t*_(205)_ −5.731. *p* ≤ 0.001; DIST = *t*_(205)_ −3.014. *p* = 003; AH = *t*_(205)_ −5.378. *p* ≤ 0.001; EE = *t*_(205)_ −6.271. *p* ≤ 0.001; DP = *t*_(205)_ −4.170. *p* ≤ 0.001 and PR = *t*_(205)_ −4.040. *p* ≤ 0.001.

These results were expected, as a previous Pearson correlation analysis showed statistically significant correlations between EE with PR (*r* = 0.649; *p* < 0.001), with REE (*r* = 0.600; *p* < 0.001), AV (*r* = 0.628; *p* < 0.001), CED (*r* = 0.568; *p* < 0.001), DIST (*r* = 0.581; *p* < 0.001) and AH (*r* = 0.550; *p* < 0.001). The correlations of PR with REE (*r* = 0.520; *p* < 0.001), VA (*r* = 0.474; *p* < 0.001), CED (*r* = 0.404; *p* < 0.001), DIST (*r* = 0.450; *p* < 0.001) and AH (*r* = 0.455; *p* < 0.001) were also statistically significant. However, the PD dimension did not show correlations with any of the PTSD variables.

In a logistic regression analysis, all the professionals who had experienced some type of aggression (physical, threats or insults) were considered as victims, regardless of the frequency with which they had suffered it. As we have seen, of the 119 professionals who experienced episodes of violence by patients in the last year, 90 experienced them on more than one occasion. Likewise, the fact of experiencing one type of violence did not exclude witnessing others. For example, of the 38 who had been physically assaulted, 31 had also received threats and 35 had been insulted.

In order to analyze the predictive role of aggressions and sociodemographic and occupational variables on different symptomatology, a multivariate logistic regression analysis was performed. A significant model was obtained with a Cox and Snell R-squared of 0.073. The only factor related to being assaulted, as shown in Table 3, was the fact of working in continuous care [*t* = 4.341, *p* ≤ 0.001; Exp(B) = 0.437].

**TABLE 3:** Multivariate analysis (logistic regression) related to being assaulted.

Victm	B	Standard error	Wald	gl	Sig	Exp(B)	95% interv Exp(B)	
							inf	sup
Interception	1.653	0.948	3.041	1	0.081			
Female sex	0.188	0.316	0.356	1	0.551	1.207	0.650	2.241
Age (25–45 years)	0.066	0.413	0.025	1	0.873	1.068	0.475	2.402
No coexistence	0.360	0.351	1.048	1	0.306	1.433	0.720	2.852
Continuous care	-0.827	0.397	4.341	1	0.037	0.437	0.201	0.952
Medical profession	-0.966	0.861	1.257	1	0.262	0.381	0.070	2.059
Nursing profession	-1.162	0.857	1.840	1	0.175	0.313	0.058	1.677
1–20 years worked	-0.179	0.426	0.177	1	0.674	0.836	0.362	1.927
Temporary contract	-0.046	0.499	0.009	1	0.926	0.955	0.359	2.538
Interim	0.619	0.384	2.607	1	0.106	1.858	0.876	3.941

Finally, linear regression analyses were performed taking each of the constituent symptoms of PTSD and the three dimensions of burnout as a dependent variable, and introducing as independent, demographic and occupational variables, as well as the different types of aggressions (Table 4).

**TABLE 4:** Linear regression model.

	Coefficient	*P*-value	Confidence interval 95%		Collinearity statistics	
			Inferior	Superior	Tolerance	VIF
**Re-experimentation**						
Constant	0.604	0.548	-1.374	2.581		
Threats	2.208	0.001	0.889	3.526	0.863	1.159
Continuous care	1.908	0.015	0.371	3.445	0.961	1.040
Physical aggressions	1.800	0.040	0.083	3.516	0.832	1.202
R2	0.140					
R2adj	0.127					
**Avoidance**						
Constant	-1.236	0.067	-2.654	0.182		
Insults	1.484	0.000	0.774	2.155	0.970	1.031
Continuous care	1.168	0.008	0.312	2.025	0.966	1.035
Sex (man)	0.866	0.015	0.171	1.561	0.995	1.005
R2	0.150					
R2adj	0.138					
**Emotional-cognitive disturbance**						
Constant	0.866	0.336	0.90	2.637		
Insults	2.165	0.000	1.207	3.122	0.872	1.147
Time in healthcare	-0.081	0.001	-0.126	-0.036	0.982	1.019
Physical aggression	2.042	0.001	0.806	3.279	0.864	1.158
Sex (man)	1.490	0.002	0.575	2.406	0.992	1.008
R2	0.272					
R2adj	0.257					
**Distancing**						
Constant	-1.494	0.074	-3.131	0.144		
Continuous attention	1.739	0.001	0.767	2.711	0.994	1.006
Threat	1.102	0.005	0.314	1.890	0.997	1.003
Sex (man)	1.059	0.010	0.259	1.859	0.996	1.004
R2	0.124					
R2adj	0.112					
**Irritation hypervigilance**						
Constant	-1.101	0.523	-2.295	4.498		
Physical aggression	3.327	0.001	1.388	5.267	0.825	1.212
Continuous care	2.973	0.001	1.227	4.720	0.943	1.061
Time in healthcare	-0.122	0.001	-0.191	-0.052	0.965	1.036
Threat	2.407	0.002	0.922	3.891	0.861	1.161
Sex (man)	1.875	0.009	0.470	3.280	0.990	1.010
R2	0.288					
R2adj	0.271					
**Emotional exhaustion**						
Constant	16.967	0.000	15.479	18.456		
Insults	5.558	0.000	3.404	7.711	0.872	1.146
Physical agress	5.019	0.000	2.250	7.788	0.872	1.146
R2	0.225					
R2adj	0.217					
**Depersonalization**						
Constant	8.356	0.000	7.820	8.892		
Threats	1.768	0.000	0.950	2.585	1.000	1.000
R2	0.081					
R2adj	0.077					
**(Lack) Personal realization**						
Constant	6.643	0.000	4.340	8.947		
Physical agress	2.070	0.002	0.790	3.351	0.841	1.189
Insults	1.248	0.013	0.270	2.226	0.872	1.147
R2	0.129					
R2adj	0.112					

Dependent variables: PTSD symptoms and burnout. Independent variables: sociodemographic, occupational and aggression variables.

The fact of having received threats from patients contributed to symptoms of re-experiencing, distancing and hypervigilance, constitutive of PTSD, as well as to the depersonalization (cynicism) dimension of burnout. Insults predicted the variables of avoidance, emotional and cognitive disturbances, emotional exhaustion and low personal realization at work. Physical aggressions were also predictive of irritation and hypervigilance symptoms and, to a lesser extent, of re-experiencing, emotional and cognitive disturbances. These types of aggressions also contributed to the emotional exhaustion and lack of realization characteristic of burnout.

The professionals working in continuous care (on shifts) were more affected by different symptoms after the aggressions than those working in ordinary care; specifically, in terms of re-experiencing, avoidance, distancing and irritation.

Although there were no statistically significant differences in terms of gender-based aggression, men who had suffered violence were more likely than women to suffer PTSD constitutive symptomatology: avoidance, emotional and cognitive alterations, distancing and hypervigilance.

The number of years in the profession showed a negative or protective relationship in terms of emotional or cognitive alterations and irritability. Likewise, the fact of practicing medicine as a profession was shown to be a protective variable in terms of realization through work.

The variables that contributed to predicting re-experiencing symptomatology (REE) were having suffered threats (*b* = 2.208; *p* = 0.001), physical aggression (*b* = 1.800; *p* = 0.040) and working in continuous care (*b* = 1.908; *p* = 0.015). VA symptoms were predicted by having received insults (*b* = 1.484; *p* ≤ 0.001), working in continuous care (*b* = 1.168; *p* = 0.008) and being male (*b* = 0.866; *p* = 0.015). Cognitive or emotional disturbances (CED) were predicted by having been a victim of insults (*b* = 2.165; *p* ≤ 0.001) or physical aggression (*b* = 2.042; *p* = 0.001), as well as being male (*b* = 1.490; *p* = 0.002), while the number of years working in healthcare appeared to be a protective variable (*b* = −0.081; *p* = 0.001). Symptoms of alienation (DIST) were contributed to by working in continuous attention (*b* = 1.739; *p* = 0.001), having experienced threats (*b* = 1.102; *p* = 0.001) and being male (*b* = 1.059; *p* = 0.010) and, finally, hypervigilance symptomatology (HA) was predicted by having experienced physical aggression (*b* = 3.327; *p* = 0.001), or threats (*b* = 2.407; *p* = 0.002), serving in continuous care (*b* = 2.973; *p* = 0.001) or being male (*b* = 1.875; *p* = 0.009).

In terms of burnout dimensions, having suffered physical aggression contributed to predicting both burnout (EE) (*b* = 5.019; *p* ≤ 0.001) and lack of Personal Accomplishment (PD) (*b* = 2.070; *p* = 0.002). Likewise, being insulted predicted the EE dimension (*b* = 5.558; *p* ≤ 0.001) as well as the lack of Personal Accomplishment (DP) (*b* = 2.070; *p* = 0.002).

## 4 Discussion

The data show not only the incidence of this phenomenon in primary care, but also the increase in this sector in recent decades (Bernaldo-De-Quirós et al., 2005; Chirico et al., 2022). To date, small health centers -with a small number of professionals and a more familiar and close treatment- had shown in several studies a lower occurrence of violent episodes than in large hospitals (Gascón et al., 2009; Mento et al., 2020). However, the most recent research confirms that no healthcare sector is free from violence. Professions that seemed immune to this phenomenon–such as radiologists, analysts, even healthcare volunteers–are also victims of aggression (Magnavita et al., 2012).

The present study did not reveal statistically significant differences between professionals who had suffered violence and those who had not in terms of gender, age, profession, seniority or administrative situation. However, as has been found in other studies, shift workers reported a higher number of assaults compared to those who worked regular hours (Lanctôt and Guay, 2014).

The results confirmed the first hypothesis: those professionals who had suffered some type of aggression showed a higher rate of psychological symptoms than those who had not. However, was not fulfilled the second hypothesis: that those who had only been victims of non-physical aggression would report a higher number of symptoms than those who had suffered physical violence.

Some theoretical and practical implications can be drawn from these results.

### 4.1 Theoretical implications

This increase in violence in primary care centers seems to have been contributed to, among other issues, by the economic crises of the last decades, the reduction in the number of professionals, the consequent increase in the pressure of care, as well as the COVID-19 pandemic and its continuous waves, which have altered relationships, favoring occasions of violence (Moleras-Serra et al., 2023).

Beyond the incidence of aggressions and vulnerability according to demographic variables, the aim was to delve into the relationship between violence suffered in the work environment and its possible symptomatology.

The first of the hypotheses was confirmed. Symptoms of stress, anxiety, constituent symptoms of PTSD, and burnout syndrome were found (Kobayashi et al., 2020). The indices in general health, measured by the GHQ-12 were moderately high considering the whole sample, and higher in the population that had experienced violence, although the difference between both groups was not statistically significant. Several studies indicate that having suffered aggression by patients is associated with an increased risk of mental health problems (Bernaldo-De-Quirós et al., 2005; Gascón et al., 2013), although the methodological diversity of the studies has yielded very different results (Lanctôt and Guay, 2014).

These incidents occurring in the healthcare setting do not usually have the magnitude of traumatic events required to cause PTSD, however, many of its symptoms can be recorded, using a non-diagnostic but approximate checklist. Those who had been victims of violent events showed a statistically significant higher incidence of PTSD-constituent symptoms. This was true for each of the subscales of “re-experiencing,” “avoidance,” “cognitive or emotional disturbances,” and “excitability and hypervigilance.” This type of symptomatology has been described after experiencing situations of violence regardless of the intensity of the violence (Kobayashi et al., 2020), with professionals experiencing at least one symptom, such as recurrent, involuntary and intrusive memories; distressing dreams; dissociative reactions; prolonged psychological distress; and intense physiological reactions when exposed to factors symbolizing the event (Lanctôt and Guay, 2014). Also, persistent efforts to avoid distressing memories and external reminders associated with the traumatic event or negative cognitive and mood alterations were reported.

The second hypothesis was partially confirmed, since both physical violence and non-physical violence were associated with psychological symptoms. In contrast to research highlighting a greater impact on psychological symptomatology in those who have suffered threats and insults (Findorff et al., 2005), in this study the impact was manifested equally in victims of physical and non-physical aggressions. It should be remembered that a high percentage of those who reported physical aggression also reported insults and threats, often repeatedly.

While physical aggressions are usually punctual and their impact may fade after a short time, verbal aggressions and threats may constitute a daily occurrence with no precise end, proving to be more deleterious on mental health (Gascón et al., 2013).

Likewise, higher and statistically significant indices were found among victims, with respect to professionals who had not suffered aggressions, in terms of the dimensions that constitute burnout syndrome: EE, DP, and lack of PR. Taking into account that this syndrome develops when there is a mismatch between work demands and the ability to cope with them effectively and understanding aggressions as a stressor over which there is little control, these could be contributing significantly to this symptomatology (Gascón et al., 2013; Viotti et al., 2015).

### 4.2 Practical implications

The centers studied have post-incident protocols which, in addition to recording the episode, provide the victim with medical, psychological and legal support. However, most were unaware of them or did not consider them useful.

It is clear that the existence of these protocols is not enough and that studies aimed at exploring possible post-incident symptomatology are necessary when designing prevention plans to mitigate the chronicity of possible harm (Lim et al., 2022). Several hospitals and primary care centers have implemented training modules aimed at healthcare workers, such as: development of resilience, self-compassion, empathy, communication skills, etc (Asensio-Martinez et al., 2019). Undoubtedly this training can be of great use for healthcare workers, but no professional can face this reality or prevent it alone. It is a structural issue of health systems, starting from zero tolerance policies.

Geoffrion et al. (2015) sought to identify individual and organizational predictors of trivialization of violence in healthcare. Overall, men were more likely to think that violence is normal in this type of work and complaining is frowned upon by peers and bosses. Healthcare organizations need to bust these myths, through information and continuing education. Similarly, reporting assaults and filing complaints are necessary actions for several reasons. Firstly, because a general registry of assaults provides a faithful map of the centers and positions most affected, allowing action to be taken on them. On the other hand, reporting a complaint deters the aggressors and reinforces the professional’s self-esteem if, in addition, they feel supported by their organization.

As we have seen, only 15.1% of those assaulted reported the incident and only one filed a complaint because the injuries were more serious. The lack of reporting is a generalized phenomenon in all countries, as confirmed in other studies and which has been explained by various reasons: lack of time, normalization of certain behaviors or even that the professionals themselves are not familiar with the prevention protocols of the centers in which they work (Spelten et al., 2020).

### 4.3 Limitations

The main limitation is that it was a retrospective study, using questionnaires, in which the professionals recalled the incidents they had experienced in the last year, as well as the feelings and symptoms that had accompanied them since then. The type of design makes it difficult to establish causal relationships; for example, the relationship between burnout and aggressions could be interpreted in a double sense. That is, that violent episodes contribute to greater emotional exhaustion and depersonalization, or that professionals who feel more burned out are found to have diminished skills for the therapeutic relationship. Likewise, the number of participants is small, and although the region in which the study was carried out is considered representative of the rest of Spain, it is difficult to generalize the results to the entire population of health professionals.

Based on this study, cross-sectional follow-up has been initiated in some of the centers studied.

## 5 Conclusion

Taking into account the results of this study, the relationships between the attacks suffered and the various subsequent symptoms are highlighted, which is very useful to undertake a more ambitious cross-sectional study over two years.

## Data availability statement

The raw data supporting the conclusions of this article will be made available by the authors, without undue reservation.

## Ethics statement

The studies involving humans were approved by the Comité de Ética en la Investigación de la Comunidad de Aragón (CEICA). The studies were conducted in accordance with the local legislation and institutional requirements. The participants provided their written informed consent to participate in this study.

## Author contributions

SG-S: Conceptualization, Funding acquisition, Investigation, Methodology, Resources, Visualization, Writing – original draft. BO-B: Formal analysis, Methodology, Validation, Writing – review & editing. MC: Conceptualization, Writing – review & editing. LS: Data curation, Writing – original draft. AA-C: Data curation, Investigation, Methodology, Writing – review & editing. YP-M: Investigation, Supervision, Writing – review & editing. PP: Data curation, Project administration, Writing – review & editing. RF-D: Formal analysis, Funding acquisition, Writing – review & editing.

## References

[B1] American Psychiatric Association (2013). *Diagnostic and statistical manual of mental disorders: DSM-5.* Washington, DC: American Psychiatric Association.

[B2] Asensio-Martinez A. Olivan-Blazquez B. Magallon-Botaya R. (2019). Relation of the psychological constructs of resilience, mindfulness, and self-compassion on the perception of physical and mental health. *Psychol .Res. Behav. Manag.* 12 1155–1166. 10.2147/PRBM.S225169 31920412 PMC6939394

[B3] Bernaldo-De-Quirós M. Piccini A. Gómez M. Cerdeira J. (2005). Psychological consequences of aggression in pre-hospital emergency care: Cross sectional survey. *Int. J. Nurs. Stud.* 52 260–270. 10.1016/j.ijnurstu.2014.05.011 24947754

[B4] Brenninkmeijer V. Van Yperen N. (2003). How to conduct research on burnout: Advantages and disadvantages of a unidimensional approach in burnout research. *Occup. Environ. Med.* 60 16–20. 10.1136/oem.60.suppl_1.i16 12782742 PMC1765718

[B5] Chirico F. Afolabi A. A. Ilesanmi O. S. Nucera G. Ferrari G. Szarpak L. (2022). Workplace violence against healthcare workers during the COVID-19 pandemic: A systematic review. *J. Health Soc. Sci.* 7:3. 10.19204/2022/WRKP2

[B6] Di Martino V. (2003). *Relationship of work stress and workplace violence in the health sector.* Geneva: International Labor Office.

[B7] Durón-Figueroa R. Cárdenas-López G. Castro-Calvo J. De Rosa-Gómez A. (2019). Adaptation of the posttraumatic stress disorder checklist for DSM-5. *Acta Invest. Psicol.* 9:3. 10.22201/fpsi.20074719e.2019.1.03

[B8] Echeburúa E. (2017). Posttraumatic stress disorder symptom severity scale according to DSM-5 criteria: Forensic version (EGS-F). *Anu. Psicol. Juríd.* 27 67–77. 10.1016/j.apj.2017.02.005

[B9] Eurofound-ETF (2022). *Living, working and COVID-19 in the European Union and 10 EU neighbouring countries.* Luxembourg: Publications Office of the European Union.

[B10] Findorff M. J. McGovern P. M. Wall M. M. Gerberich S. G. (2005). Reporting violence to a health care employer: A cross-sectional study. *AAOHN JOURNAL* 53, 399–406. 16193912

[B11] Gascón S. Leiter M. P. Andrés E. Martínez-Jarreta B. (2013). The role of aggressions suffered by healthcare workers as predictors of burnout. *J. Clin. Nurs.* 22 3120–3129. 10.1111/j.1365-2702.2012.04255.x 22978353

[B12] Gascón S. Martínez-Jarreta B. González-Andrade J. F. Rueda M. Á (2009). Aggression towards health care workers in Spain: A multi-facility study to evaluate the distribution of growing violence among professionals, health facilities and departments. *Int. J. Occup. Environ. Health* 15 29–35. 10.1179/107735209799449707 19267124

[B13] Geoffrion S. Lanctôt N. Marchand A. Boyer R. Guay S. (2015). Predictors of trivialization of workplace violence among healthcare workers and law enforcers. *J. Threat Assess. Manag.* 2:195. 10.1037/tam0000048

[B14] Hair J. Black W. Babin B. Anderson R. Tatham R. (2006). *Multivariate data analysis.* Upper Saddle River, NJ: Pearson Prentice-Hall.

[B15] Kind N. Eckert A. Steinlin C. Fegert J. M. Schmid M. (2018). Verbal and physical client aggression – a longitudinal analysis of professional caregivers’ psychophysiological stress response and burnout. *Psychoneuroendocrinology* 94 11–16. 10.1016/j.psyneuen.2018.05.001 29738960

[B16] Kobayashi Y. Oe M. Ishida T. Matsuoka M. Chiba H. Uchimura N. (2020). Workplace violence and its effects on burnout and secondary traumatic stress among mental healthcare nurses in Japan. *Int. J. Environ. Res. Public Health* 17:2747. 10.3390/ijerph17082747 32316142 PMC7215457

[B17] Lanctôt N. Guay S. (2014). The aftermath of workplace violence among healthcare workers: A systematic literature review of the consequences. *Aggress. Violent Behav.* 19 492–501. 10.1016/j.avb.2014.07.010

[B18] Lim M. C. Jeffree M. S. Saupin S. S. Giloi N. Lukman K. A. (2022). Workplace violence in healthcare settings: The risk factors, implications and collaborative preventive measures. *Ann. Med. Surg.* 78:103727. 10.1016/j.amsu.2022.103727 35734684 PMC9206999

[B19] Magnavita N. Fileni A. Pescarini L. Magnavita G. (2012). Violence against radiologists. I: Prevalence and preventive measures. *Radiol. Med.* 117 1019–1033. 10.1007/s11547-012-0825-7 22580806

[B20] Maslach C. Leiter M. P. (2008). Early predictors of job burnout and engagement. *J. Appl. Psychol.* 93 498–512. 18457483 10.1037/0021-9010.93.3.498

[B21] Maslach C. Leiter M. P. (2016). “Burnout,” in *Fink Concepts, cognition, emotion, and behaviour*, ed. Stress G. (Cambridge, MA: Academic Press), 351–357.

[B22] Menckel E. Viitasara E. (2002). Threats and violence in Swedish care and welfare–magnitude of the problem and impact on municipal personnel. *Scand. J. Car. Sci.* 16 376–385. 10.1046/j.1471-6712.2002.00103.x 12445107

[B23] Mento C. Silvestri M. C. Bruno A. Zoccali R. A. (2020). Workplace violence against healthcare professionals: A systematic review. *Aggress. Violent Behav.* 51:101381. 10.1016/j.avb.2020.101381

[B24] Moleras-Serra A. Morros-Pedros R. Monteagudo M. Gómez-Lumbreras A. (2023). Primary health care research in COVID-19: Analysis of the protocols reviewed by the ethics committee of IDIAPJGol, Catalonia. *BMC Prim. Care* 24:91. 10.1186/s12875-023-02025-5 37024845 PMC10078015

[B25] Rudkjoebing L. A. Bungum A. B. Flachs E. M. Eller N. H. Borritz M. Aust B. (2020). Work-related exposure to violence or threats and risk of mental disorders and symptoms: A systematic review and meta-analysis. *Scand. J. Work Environ. Health* 46:339. 10.5271/sjweh.3877 31909816 PMC8506313

[B26] Salanova M. Schaufeli W. B. Llorens Gumbau S. Silla P. Grau Gumbau R. M. (2000). Desde el burnout al engagement: Una nueva perspectiva? *J. Work Organ. Psychol.* 16 117–134.

[B27] Sánchez-López M. Dresch V. (2008). The 12-item general health questionnaire (GHQ-12): Reliability, external validity, and factor structure in the Spanish population. *Psicothema* 20 839–843. 18940092

[B28] Schaufeli W. B. Bakker A. Schaap C. Kladler A. Hoogduin C. A. L. (2001). On the clinical validity of the Maslach Burnout Inventory and the Burnout Measure. *Psychol. Health* 16 565–582. 22804499 10.1080/08870440108405527

[B29] Soberón C. Crespo M. Gómez-Gutiérrez M. D. M. Armour C. (2016). Dimensional structure of DSM-5 posttraumatic stress symptoms in Spanish trauma victims. *Eur. J. Psychotraumatol.* 7:32078. 10.3402/ejpt.v7.32078 27974133 PMC5156862

[B30] Spelten E. Thomas B. O’Meara P. F. Begg S. J. (2020). Organisational interventions for preventing and minimising aggression directed towards healthcare workers by patients and patient advocates. *Cochrane Database Syst. Rev.* 4:CD012662. 10.1002/14651858.CD012662 PMC7197696 32352565

[B31] The Health Division of Safety Research (1996). *Violence in the workplace: Risk factors and prevention strategies.* Morgantown, WV: Health Division of Safety Research.

[B32] Varghese A. Joseph J. Vijay V. R. Khakha D. C. Dhandapani M. Gigini G. (2022). Prevalence and determinants of workplace violence among nurses in the South-East Asian and Western Pacific regions: A systematic review and meta-analysis. *J. Clin. Nurs.* 31 798–819. 10.1111/jocn.15987 34351652

[B33] Viotti S. Gilardi S. Guglielmetti C. Converso D. (2015). Verbal aggression from care recipients as a risk factor among nursing staff: A study on burnout in the JD-R model perspective. *Biomed Res. Int.* 2015:215267. 10.1155/2015/215267 26568956 PMC4628963

[B34] Weathers F. W. Litz B. T. Keane T. M. Palmieri P. A. Marx B. P. Schnurr P. P. (2013). *The ptsd checklist for dsm-5 (pcl-5).* Washington, DC: U.S. Department of Veterans Affairs.

[B35] Winstanley S. Whittington R. (2004). Aggression towards health care staff in a UK general hospital: variation among professions and departments. *J. Clin. Nurs.* 13 3–10. 10.1111/j.1365-2702.2004.00807.x 14687287

